# Following the Majority: Social Influence in Trusting Behavior

**DOI:** 10.3389/fnins.2019.00089

**Published:** 2019-02-11

**Authors:** Zhenyu Wei, Zhiying Zhao, Yong Zheng

**Affiliations:** ^1^Key Laboratory for NeuroInformation of Ministry of Education, School of Life Science and Technology, University of Electronic Science and Technology of China, Chengdu, China; ^2^Key Laboratory of Cognition and Personality (MOE), Faculty of Psychology, Southwest University, Chongqing, China

**Keywords:** social influence, trust game, superior temporal gyrus, ventral striatum, reward learning

## Abstract

When making decisions, people may change their behavior, sometimes against their personal preference, according to the opinions of peers. However, the effect of social influence on trust is still unknown. In our study, we used the event-related functional magnetic resonance imaging to investigate brain activity in social influence during a trust game. The behavioral results revealed that people tend to conform to others’ opinions and behaviors in a trust game. Decreased activations were observed in superior temporal gyrus during processing of social influences. Moreover, brain regions supporting value processing and reward learning were activated when subjects decided to follow the majority. These regions include the ventral medial prefrontal cortex, ventral striatum, and parahippocampal gyrus. Finally, our exploratory analysis revealed an increase in functional connectivity between the prefrontal cortex and the ventral striatum during conformity in trusting behavior. These findings indicate that the neural basis of social influence in trusting behavior are similar to the mechanisms implicated in reward learning. The brain regions involved in reward learning might reflect the reward value of agreeing with others in our study.

## Introduction

Our opinions and behaviors are often affected by the majority (Asch, 1956; Turner, 1991). People tend to change their opinions and behaviors in order to follow with social norms, even if the majority decision is against their personal preference (Cialdini and Goldstein, 2004; Morgan and Laland, 2012; Haun et al., 2013). Psychologists defined this phenomenon as “social conformity.” It refers to individuals’ action of adopting the opinions, behaviors, and judgments of others (Turner, 1991). Asch (1951) used a simple line judgment task to investigate social conformity. Since then social psychologists began to explore the causes of social conformity. Based on previous study, there are three types of intrinsic motivations underlying social conformity: a desire to obtain social approval of others, a desire to make a correct choice, and a desire to keep a positive self-concept (Cialdini and Goldstein, 2004).

Recent studies have investigated the effect of conformity in many judgment tasks as well as the neural basis of conformity. By using mental rotation task and music rating task, Berns et al. (2005, 2010) found that the opinions of peers could change participants’ initial judgments and affect neural activity within relatively low-level processing brain areas related to each task. In addition, previous literatures have reported that the brain regions associated with reward processing and behavioral adjustment were closely associated with social influence. Mason et al. (2009) exposed subjects to popular, unpopular and novel symbols and reported that the medial prefrontal cortex (mPFC) was involved in normative social influence by comparing socially and not socially marked symbols, while the striatum (the caudate) might be a possible index of informational social influence by comparing popular and unpopular symbols. Wei et al. (2013) also found that confliction with group norms during an ultimatum game activated the bilateral insula, bilateral middle frontal gyrus (MFG) and mPFC. Additionally, Klucharev et al. (2009) found that conflicting group opinions triggered a neuronal response in the nucleus accumbens and the rostral cingulate zone (RCZ). These brain regions are often associated with reward processing and behavioral adjustment, which is similar to prediction error signal (Berns et al., 2001; Holroyd and Coles, 2002; Ridderinkhof et al., 2004). Neural activity in these regions could predict participants’ subsequent conforming behaviors (Klucharev et al., 2009). By using stock task and music choice task, Burke et al. (2010) and Campbell-Meiklejohn et al. (2010) found that neural activity in the ventral striatum was involved in social influence, suggesting that the opinions of others could modulate the basic value signals in known reinforcement learning neural circuitry (Campbell-Meiklejohn et al., 2010).

Conformity effect was also found in economic decisions, such as ultimatum game (Wei et al., 2013), dictator game (Wei et al., 2017), risk taking (Gardner and Steinberg, 2005), stock market participation (Hong et al., 2004), consuming decision and investment decision (Bursztyn et al., 2014). These results indicated that the opinion of majority could influence people’s own preferences in economic decision context. Trust plays an important role in economic decision interactions (Cochard et al., 2004). Previous study suggested that, for the trusting behaviors, genetics only explain about 20% of the cross-sectional variation while environmental factors would explain 80% of the variation (Cesarini et al., 2008; Ahern et al., 2014). One potential environment factor is social conformity. Prior studies have found that individuals tended to change their rating of trustworthiness toward social norm in a trustworthiness judgment task (Campbell-Meiklejohn et al., 2012; Simonsen et al., 2014). In present study, we used trust game to explore whether peers’ decision could change the choices of individuals. Trust game is widely used to measure trusting behavior. There are two players in the classic trust game: an investor and a trustee. Both players are endowed with $10. The investor decides whether give the money to the trustee. If the investor gives the money to the trustee, the endowment would be multiplied by experimenter then. In the end, the trustee decides whether to give any portion of the money she/he received back to the investor or just keep it. In our study, we developed a modified trust game. In this task, participants were able to see peer’ choices when they made the trust decision.

Firstly, we hypothesized that the choices of the majority would affect subjects’ trust preference. Subjects may invest the money to the trustee when they see that the majority of the group trusts the trustee. Conversely, participants may distrust the trustee if they see that the majority does not trust the trustee. Otherwise, subjects will insist on their own trust preferences if social influence has no effects on trust decision. Secondly, we predicted that participants may conform to the opinion of the majority with a relatively high level of decision confidence, since they may have high reward expectancy in the trust social influence condition. Finally, previous literatures had reported that social influence might affect participants’ behaviors through the neural underpinnings of reward learning and behavioral adjustment, such as ventral medial prefrontal cortex (vmPFC) and anterior cingulate cortex (ACC), and also brain structures underlying social reward processing especially the striatum (Izuma et al., 2008; Mason et al., 2009; Klucharev et al., 2011; Wei et al., 2013). Therefore, we hypothesized that the activity in brain reward circuits such as the vmPFC and caudate may be associated with social influence. Recent brain imaging studies have suggested evidence that enhanced functional connectivity between the prefrontal cortex and ventral striatum during reward processing (Camara et al., 2008). Hence, we hypothesized that a psychophysiological interaction (PPI) analysis may confirm an enhanced functional connectivity between the prefrontal cortex and ventral striatum during conformity in the trust social influence condition.

## Materials and Methods

### Participants

Twenty-seven healthy right-handed participants (mean age = 21.1, female = 16, male = 11) participated in the experiment. These participants were recruited from Southwest University through advertisements in the online student forums, none of them came from department of psychology or economics. All were native Mandarin speakers, with no neurological illness as confirmed by psychiatric clinical assessment or psychological disorders, and with (corrected to) normal vision. Written informed consent was obtained in accordance with the regulations of the Ethics Committee of Southwest University. This study was approved by the Ethics Committee of Southwest University.

### Stimulus Materials

Peers’ choices were presented in the form of a table to the participants. The number “1” refers to a choice to send the endowment to the stranger and the number “2” indicates a choice to keep the endowment. There were four conditions of social influence: trust influence (three or four group members decided to send the endowment to the stranger); moderate (two group members decided to trust the stranger while the other two decided keep their endowments); distrust influence (three or four group members decided to keep the endowment); and no information (the boxes corresponding to each group members’ choices were replaced with “×”). There were 70 offers in total. The offer stimuli consisted of the number of the trustee (randomly from 1 to 70), the choices available, and the social information (peers’ choices). The former was presented in the upper portion of the screen. The choices available were presented in the center of the screen and the latter in the lower part of the picture.

### Experimental Procedures

Participants were told that they would play an on-line monetary game with four other participants, who would be in a separate behavioral laboratory. They would see the choices of the other peers on the computer screen during the decision phase of the experiment. Participants acted as an investor and play the game independently with 70 different strangers (trustees). These trustees were randomly selected from the university and played the game on the other floor. Participants and their group members did not know anything about these seventy trustees. At the beginning of each trial, both players (investor and trustee) were endowed with ¥10. The investor was asked to decide whether to send the endowment. The endowment would be tripled if the investor decided to invest. Then the trustee was asked to decide whether to send half of the money back (¥15). The investor would not know the outcome (i.e., trustees’ choice) during the task. Subjects were told that they will receive ¥50 for participating in the experiment plus the additional money earned from ten of their trust decisions, chosen at random, in the trust game. Subjects earned on average about ¥60 for their participated in the experiment which was not based on investment outcome. We asked participants whether she/he believed the existence of trustees after they finished the task. All the participants reported that they believed the existence of trustees. After the data of all the participants were collected, participants received payment and were told that the peers and trustees did not exist.

**FIGURE 1:**
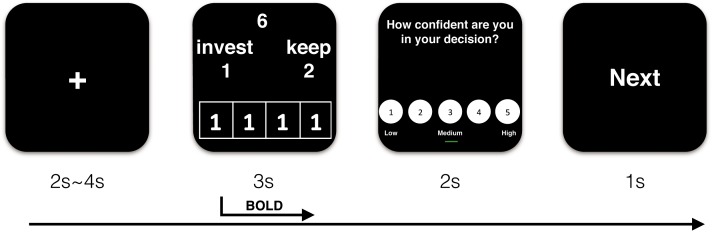
Demonstration of sequence of events in a trial (take trust influence condition for example).

Participants then received details about the procedure of the experiment. At the beginning of each trial, they saw a fixation point for a 2–4 s jittered duration that varied pseudo-randomly. Then, the decision screen was presented for 3 s. They used the index and middle fingers of their right hands to separately respond to the offer by pressing one of two buttons on an MRI-compatible button box (“1” to invest and “2” to keep the endowment). Peers’ choices were placed in the lower part of the decision interface. Subsequently, confidence ratings were provided for 2 s. Finally, the word “next” displayed for 1 s, indicating that the next trial was about to begin. The sequence of events in a trial is illustrated in Figure 1.

There were seventy trials in present experiment. The duration of a trial is approximately 9 seconds. In 10 of the trials, participants were informed that two peers decided to send the money to the trustee while the other two decided to keep the endowments. These trials were used solely to maintain the believability of the interaction between the participant and the four peers. They were excluded in the final analysis. In one-third of the remaining trials (20 trials), participants could not see the group’s choices (the no information, or baseline condition; we told participants that the decisions in these trials were not made by all the four peers). For the 20 trials of the trust influence condition, three or four peers’ choices were to send the endowments to the trustee. For the 20 trials of the distrust influence condition, one or none of the group members decided to invest. Before performing the task in the scanner, all participants completed a training session. They were told that the computer for the pre-experiment training is not connected to the local network, therefore they could not receive anything information about the peers’ choices.

We used a PC running E-Prime 2.0 to display the stimuli and acquire the responses of the participants, as well as the reaction times (RTs). In the scanner, there was a mirror placed on the top of the image acquisition coil. Participants saw the experiment task via this mirror that reflected the screen mounted at the back of the scanner.

### Image Acquisition

Functional MRI data were acquired using a 3T Siemens Trio scanner. Each scan contains 355 functional volumes, using an echo-planar imaging (EPI) sequence with the following parameters: TR/TE = 2000/30 ms, flip angle = 90°, acquisition matrix = 64 × 64, FOV = 192 mm × 192 mm, axial slices = 32, slice thickness/gap = 3mm/1 mm, voxel size = 3 mm × 3 mm × 3 mm. The first three images were discarded for the saturation effect.

### Data Analysis

#### Behavioral Data Analysis

We used statistical product and service solutions (SPSS) to analyze the behavioral data. We predicted that the choices of the majority may influence participants’ decision. A repeated measure (social influence: baseline, trust influence, distrust influence) ANOVA was used to analyze the RTs in the decision phase, as well as the rate of trust. Since we predicted that subjects may have high reward expectancy in the trust social influence condition, we conducted a 3 (social influence: baseline, trust influence, distrust influence) × 2 (choices: trust, distrust) ANOVA on the mean confidence rating.

#### fMRI Data Analysis

Image preprocessing was performed with statistical parametric mapping 8 (SPM8; Welcome Department of Imaging Neuroscience, University of London, United Kingdom). Functional images were first corrected for motion artifacts. Then images were interpolated to correct for slice timing, and spatially normalized into the Montreal Neurological Institute (MNI)-space using the SPM8 EPI template, and resampled into 3 mm × 3 mm × 3 mm voxels. Images were smoothed using an 8 mm^3^ full-width-at-half-maximum (FWHM) Gaussian kernel. A 0.01 Hz–0.08 Hz band-pass filter, which was composed of a discrete cosine-basis function with a cutoff period of 128 s for the high-pass filter was applied to the time courses of all brain voxels.

We conducted analysis on functional magnetic resonance imaging data of the decision phase. General linear model analysis was performed with SPM8. Three regressors were entered based on social information (baseline, trust influence and distrust influence). These regressors were then convolved with the standard hemodynamic response function. In addition, the realignment parameters were included in the model to regress out potential movement artifacts. For a whole-brain analysis, the result was thresholded at *p* < 0.05 (FDR correction), cluster size > 10. The effect of social influence was estimated by contrasting the trust influence effect (*trust influence condition* > *no information*). For more detailed insights into the neural mechanisms underlying social conformity in trusting behavior, we did an exploratory analysis, analyzed the conforming behavior contrast (*conformity vs. non-conformity*) in trust influence condition (*trust influence condition – conformity* > *trust influence condition – non-conformity*). Activations in this analysis were thresholded at *p* < 0.05 (FDR correction), cluster size > 10.

Finally, an exploratory PPI analysis was performed in order to identify brain regions that showed significantly increased coordination (i.e., increased functional connectivity) with the ventral striatum activity related to conformity compared to non-conformity in the trust influence condition (Friston et al., 1997). Based on our fMRI results and previous literature, the region of interest (ROI) was defined as a sphere with 6-mm-radius centered at the peak voxel in the ventral striatum (MNI coordinates: [10, 18, -9]) (Campbell-Meiklejohn et al., 2010). The time series was extracted from each subject in the ventral striatum. And the PPI regressor was calculated as the element-by-element product of the mean-corrected activity of ROI and a vector coding for differential task effects of conformity-trust influence versus non-conformity-trust influence. The PPI regressors reflected the interaction between psychological variable (*trust influence condition - conformity* > *trust influence condition – non-conformity*) and the activation time course of the ventral striatum. Individual contrast images for conformity-trust influence versus non-conformity-trust influence were computed and entered into second-level one-sample *t*-tests. Brain regions surviving the cluster-extent based threshold *p* < 0.05 (FDR correction, with a primary voxel-level threshold of *p* < 0.001) were considered significant.

## Results

### Behavioral Results

Data from twenty-seven subjects entered the behavioral analysis. We used a one-way repeated measures (social influence: baseline, trust influence, distrust influence) ANOVA to analyze the RTs in the decision phase. The effect of social influence was significant, *F*(2,25) = 4.204, *p* < 0.05. Participants responded faster in the trust influence condition (*M* = 1222.46 ms, *SD* = 312.76) than in the baseline condition (*M* = 1328.58 ms, *SD* = 333.87), *t*_(26)_ = -2.845, *p* < 0.01. The responses were also faster in the trust influence condition (*M* = 1222.46 ms, *SD* = 312.76) than in the distrust condition (*M* = 1294.6 ms, *SD* = 294.42), *t*_(26)_ = -2.479, *p* < 0.05.

Regarding the subjects’ choices, a one-way repeated measures (social influence: baseline, trust influence, distrust influence) ANOVA was used to analyze the rate of trust in the decision phase. The effect of social influence was significant, *F*(2,25) = 7.714, *p* < 0.01. Subjects decided to trust the trustee at a significantly higher rate in the trust influence condition (*M* = 0.72, *SD* = 0.2) than in the baseline condition (*M* = 0.53, *SD* = 0.22), *t*_(26)_ = 3.543, *p* < 0.01. We also found this phenomenon in the contrast between trust influence condition (*M* = 0.72, *SD* = 0.2) and distrust influence condition (*M* = 0.43, *SD* = 0.27), *t*_(26)_ = 3.926, *p* < 0.001. Participants chose to trust the trustee at a significantly higher rate in the baseline condition (*M* = 0.53, *SD* = 0.22) than in the distrust influence condition (*M* = 0.43, *SD* = 0.27), *t*_(26)_ = 2.074, *p* < 0.05.

Because we predicted that subjects may have high reward expectancy in the trust social influence condition, we hypothesized that participants may conform to the opinion of the majority with a relatively high level of decision confidence. We conducted a 3 (social influence: baseline, trust influence, distrust influence) × 2 (choices: trust, distrust) ANOVA on the mean confidence rating. As predicted, the interaction between social influence and choices was significant, *F*(2,25) = 9.202, *p* < 0.001. The level of decision confidence is higher in the trust influence-trust condition (*M* = 3.78, *SD* = 0.52) than in the baseline-trust condition (*M* = 3.44, *SD* = 0.83), *t*_(26)_ = 2.632, *p* < 0.05, as well as in the distrust influence-trust condition (*M* = 3.37, *SD* = 0.74), *t*_(26)_ = 3.227, *p* < 0.01. Confidence ratings for the trust influence-trust condition (*M* = 3.78, *SD* = 0.52) seemed to be overall higher than ratings for the trust influence-distrust condition (*M* = 3.37, *SD* = 0.61), *t*_(26)_ = 3.827, *p* < 0.001.

### fMRI Results

**FIGURE 2:**
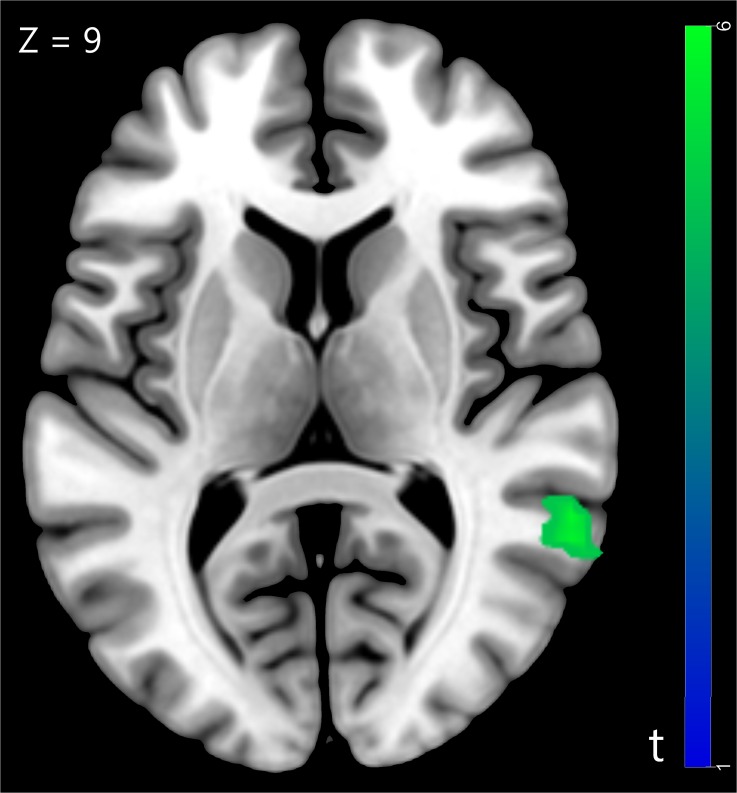
The superior temporal gyrus was involved in trust influence condition (Trust influence > Baseline), *p* < 0.05, cluster size = 10, FDR correction.

We compared the neural activity in trust influence condition with baseline condition and found significantly greater deactivation in superior temporal gyrus (STG) (for more details see Table 1 and Figure 2). The STG is a key brain region that involved in the cognitive capacity of perspective taking (Frith and Frith, 2003).

**Table 1:** Significant activation clusters for trust social influence.

					No. of
Brain regions	HEM	x	y	z	voxels	*t*-value
**Trust influence > Baseline**						
*Activation*						
No Cluster						
*Deactivation*						
STG	R	60	-45	9	28	5.97


*Voxels were selected for p < 0.05, cluster size > 10, FDR correction. HEM, hemisphere; STG, superior temporal gyrus.*

**FIGURE 3:**
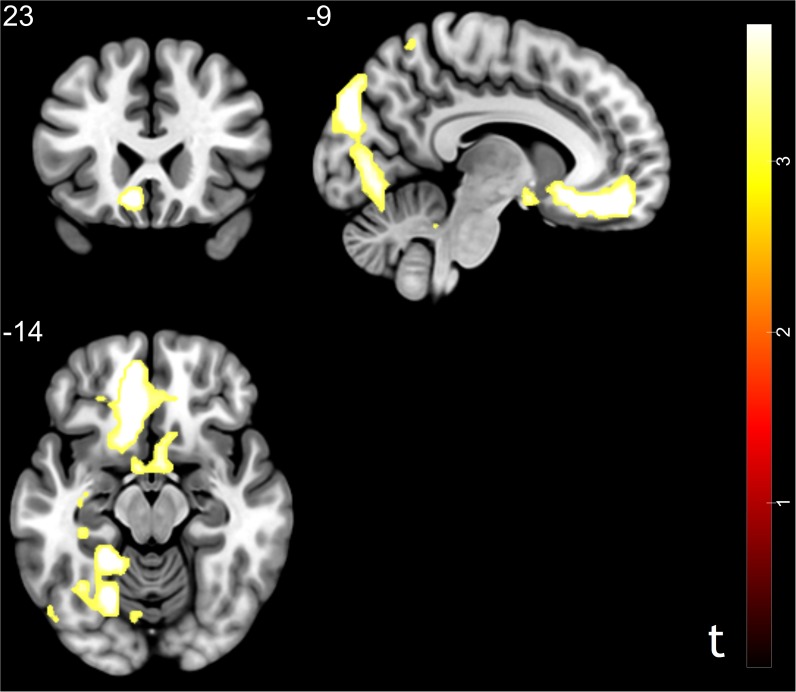
Brain regions correlated with social influence in trusting behavior (trust influence – conformity > trust influence – non-conformity). Significant activations in middle frontal gyrus, middle temporal gyrus, middle occipital gyrus, rostral cingulate zone, anterior cingulate cortex, ventral medial prefrontal cortex, and inferior parietal lobule. *p* < 0.05, cluster size = 10, FDR correction.

To capture the neural mechanisms underlying conformity effect in trusting behavior, exploratory analyses were performed. We compared the trust influence-conformity trials (mean number of trials 14) to trust influence-non-conformity (mean number of trials 6). Results shown that the trust influence which successfully induced conformity in trusting behavior activated the brain regions such as bilateral parahippocampal gyrus, vmPFC, RCZ, ACC/ caudate, middle occipital gyrus (MOG), MFG, middle temporal gyrus (MTG), postcentral gyrus and inferior parietal lobule (IPL) (see Table 2 and Figure 3 for more details). Comparison of activity in non-conformity trials with conformity trials did not show any significant activation.

**Table 2:** Significant activation clusters for conformity in trusting behavior.

					No. of
Brain regions	HEM	x	y	z	voxels	*t*-value
MFG	L	-36	42	42	16	3.8
MTG	R	60	-63	-9	29	4.9
MOG	L	-51	-81	3	29	3.98
RCZ	R	3	-3	39	23	3.77
ACC/Caudate	L	-9	27	-18	43	5.64
vmPFC	L	-6	51	-18	137	5.36
IPL	R	57	-30	30	32	3.92
Postcentral gyrus	R	60	-12	48	210	4.88
Parahippocampal gyrus	L	-9	-87	30	11	5.4
Parahippocampal gyrus	R	39	-6	-36	38	7.96


*Voxels were selected for p < 0.05, cluster size > 10, FDR correction. HEM, hemisphere; MFG, middle frontal gyrus; MTG, middle temporal gyrus; MOG, middle occipital gyrus; RCZ, rostral cingulate zone; ACC, anterior cingulate cortex; vmPFC, ventral medial prefrontal cortex; IPL, inferior parietal lobule.*

**FIGURE 4:**
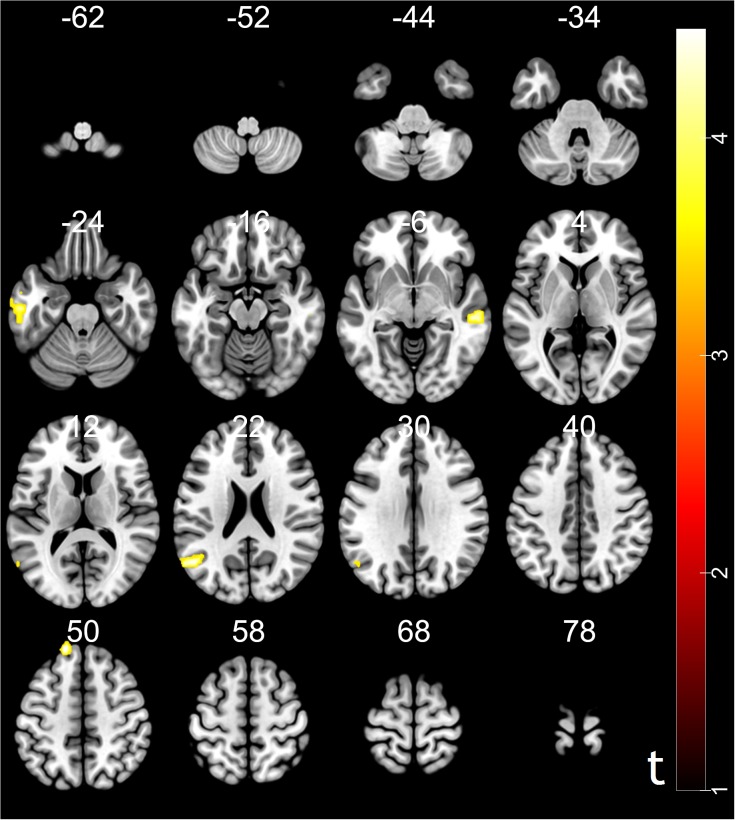
Results of psychophysiological interaction (PPI) analysis. The region of interest was ventral striatum, MNI coordinates: [10, 18, –9]. Functional connectivity with the ventral striatum (conformity > non-conformity) in the trust influence condition. Voxels were selected for *p* < 0.05, FDR cluster-level correction with an initial peak-level threshold *p* < 0.001.

Moreover, psychophysiological interaction (PPI) analysis showed that activity in the ventral striatum was accompanied by task-dependent (conformity > non-conformity) functional interaction with brain areas: STG, superior frontal gyrus (SFG), MTG and inferior temporal gyrus (ITG). The opposite contrast did not reveal any significant changes in functional connectivity (see Table 3 and Figure 4 for more details).

**Table 3:** Results of psychophysiological interaction (PPI) analysis.

					No. of
Brain regions	HEM	x	y	z	voxels	*t*-value
STG	L	-51	-63	21	87	4.74
SFG	L	-18	48	51	48	4.83
MTG	R	57	-24	-9	51	5.16
ITG	L	-57	-18	-27	53	4.78


*Voxels were selected for p < 0.05, FDR cluster-level correction with an initial peak-level threshold p < 0.001. HEM, hemisphere; STG, superior temporal gyrus; SFG, superior frontal gyrus; MTG, middle temporal gyrus; ITG, inferior temporal gyrus.*

## Discussion

In the present study, we used psychological and neuroscientific methods to investigate the impact of social influence on trust. We found that individuals are likely to conform to the opinions of their peers in a trust game. The rate of trust was higher when participants found that the majority of group members trusted the trustee compared to in the baseline condition. Conversely, the rate of trust was lower when participants saw that most group members decided to keep the endowment (distrust) compared to in the baseline condition. In addition, participants conformed to the opinion of the majority with relatively high levels of decision confidence in the trust influence condition.

Functional imaging data suggested that the STG, a brain region involved in perspective-taking, was decreased when participants made decision in the trust influence condition comparing with the baseline condition. The activity of STG is associated with perspective taking, which can be termed as theory of mind (Frith and Frith, 2003). As the decision to trust is concerned with perspective-taking, it should activate brain regions involved in theory-of-mind tasks (Fehr and Camerer, 2007). Moreover, researchers found the STG was involved in the processing of gaze direction in a modified trust game (Sun et al., 2018). A previous study that focused on the neurobiological correlates of conformity during mental rotation task has reported that the presence of external information was associated with decreased activation in the mental rotation neural network (Berns et al., 2005). They inferred that the external information relieved the mental rotation processing load (Berns et al., 2005). Similarly, decreased activations were observed during trust game in STG when external information was presented in our study. This result might suggest that external trust information affected neural activity in brain regions associated with trust game, which relieved the perspective-taking process in the game.

In our study, we tried to capture the conformity effect in the imaging data and found that brain regions involved in reward learning such as the vmPFC, ACC, ventral striatum, parahippocampal gyrus, and RCZ were also related with social influence in trusting behavior. The vmPFC has been previously implicated in processing reward expectations and computing the subjective value of multiple reward types (Rushworth et al., 2009, 2011; Rangel and Hare, 2010; Grabenhorst and Rolls, 2011). The study of brain activity during decision-making suggested that fictive reward signals (rewards that could have been, but were not directly received) have been represented in the ACC (Hayden et al., 2009). The RCZ is engaged when the need for adjustments to achieve action goals becomes evident (Ridderinkhof et al., 2004). Previous studies have demonstrated that the caudate is involved in gain prediction in response to reward cues and implicated in reward processing, social learning, and reciprocate cooperation (Rilling et al., 2002, 2004; McCoy and Platt, 2005; Knutson and Wimmer, 2007). According to PPI results, we found possible enhanced functional connectivity between the ventral striatum and prefrontal cortex during conformity compared to non-conformity in trusting behavior. Notably, recent research demonstrated that increased functional connectivity between the ventral striatum and prefrontal cortex was related to reward processing (Frank and Claus, 2006; Camara et al., 2008, 2009; van den Bos et al., 2012). Taken together, these exploratory imaging results suggest that the underlying mechanisms of social influence in trusting behavior may be similar to those implicated in reward learning. Agreement with the other group members might predict future acceptance from peer, which can also activate the reward system (Izuma and Adolphs, 2013). These exploratory findings were consistent with the results of previous studies that reported that social influence effect affects participants’ behaviors through the neural mechanisms involved in reward learning and behavioral adjustment (Izuma et al., 2008; Mason et al., 2009; Wei et al., 2013).

Several limitations of this study should be noted. Firstly, the present task is different from the Asch’s experiment. In our study, subjects had no other information about trust decision except the group members’ choices. This manipulation can potentially lead to conforming to the group member. Secondly, we did not use scale to quantitatively measure whether participants believed the experiment manipulation, which might also affect the result. Thirdly, the number of non-conformity trials that were included in exploratory analysis was less than 10 which limited the power of our GLM model. Despite that the results for these analyses survived correction, further studies could consider increasing the number of trials in order to more reliably evaluate these effects.

## Conclusion

The present study provides evidence of the relationship between social influence and trust decisions. It complements previous research by assessing the neural basis of social influence and extends our understanding of the decision to trust. Our behavioral results revealed that individuals are likely to be influenced by others’ opinions and conform to the opinions of peers in a trust game. Participants conformed to the opinion of the majority with a relatively high level of decision confidence as a result of the high reward expectancy in the trust social influence condition. Decreased activations were observed in STG when external information was presented and this result might suggest that external trust information affected neural activity in brain regions associated with trust game, which relieved the perspective-taking process in the trust game. The results of exploratory analysis indicated that the brain regions involved in value processing and reward learning, such as the vmPFC, ventral striatum, ACC, and parahippocampal gyrus, were activated when subjects decided to follow the majority in trusting behavior. The PPI analysis confirmed possible increased functional connectivity between the ventral striatum and the prefrontal cortex during conformity in trusting behavior. In conclusion, these findings suggest that the mechanisms underlying social influence in trusting behavior may be similar to those implicated in reward learning.

## Author Contributions

ZW and YZ conceived and designed the experiments. ZW and ZZ programed the task and analyzed the data. ZW performed the experiments. ZW, ZZ and YZ wrote the paper.

## Conflict of Interest Statement

The authors declare that the research was conducted in the absence of any commercial or financial relationships that could be construed as a potential conflict of interest.
